# Simple detection of gluten in commercial gluten‐containing samples with a novel nanoflower electrosensor made of molybdenum disulfide with comparison of the ELISA method

**DOI:** 10.1111/1750-3841.17043

**Published:** 2024-04-02

**Authors:** Fırat Salman, Adem Zengin, Hilal Çelik Kazici

**Affiliations:** ^1^ Department of Chemical Engineering, Faculty of Engineering Van Yüzüncü Yıl University Van Turkey; ^2^ Department of Chemistry Faculty of Science Van Yüzüncü Yıl University Van Turkey

**Keywords:** differential pulse voltammetry, electrochemical sensor, gluten, MoS_2_ nanoflowers

## Abstract

In this study, a new electrochemical sensor based on molybdenum disulfide (MoS_2_) nanoflowers/glassy carbon electrode (GCE was created for the sensitive detection of gluten. The prepared nanocatalysts were characterized using scanning electron microscopy with energy dispersive spectroscopy, x‐ray diffraction, and x‐ray photoelectron spectroscopy. The effects of the prepared nanocatalysts, pH value, and dropping amounts on the results were examined in detail. The electrochemical performance of the developed sensor (MoS_2_ nanoflowers/GCE) was then evaluated using differential pulse voltammetry, and the sensor was found to have significant electrochemical activity against gluten. A substantial linear connection was observed in the range of 0.5–100 ppm of gluten concentration under optimum experimental circumstances, and the detection limit between peak current and gluten concentration was determined as 1.16 ppm. The findings showed that the MoS_2_ nanoflowers/GCE gluten sensor has exceptional selectivity and stability. Finally, the generated electrochemical sensor was effectively utilized for gluten detection in commercial gluten‐containing materials with a detection limit of 0.1652 ppm. Thus, the developed MoS_2_ nanoflowers/GCE sensor offers a potential method for the detection of other molecules and is a promising candidate for gluten detection in commercial samples.

## 1 INTRODUCTION

Celiac disease (CD) is a multi‐systemic autoimmune disorder caused by gluten protein components present in grains such as wheat, oats, rye, and barley in genetically predisposed individuals. The symptoms of this disease were first described in 1887 as growth retardation, diarrhea, and exhaustion. In 1953, it was determined that grains such as wheat, oats, rye, and barley cause celiac disease (Van Bergeijk et al., 1993). Gluten proteins (which can be found in wheat, rye, and barley) that cause celiac disease are classified as gluten, gliadin, hordein, and secalin (Niewinski, 2008). Proteases in the upper section of the digestive tract are unable to completely break down gluten proteins, thus staying intact in the intestinal lumen. In celiac patients, the enzyme transglutaminase deaminates gluten protein fractions in the upper small intestine. This structure causes villous damage, as well as the release of other tissue‐damaging factors. As a result, the surface epithelium is harmed, and the villi (sucking feathers) become flattened (Bethune & Khosla, 2008; Catassi & Fasano, 2008). When villi are damaged, the absorption and digestion of foods cannot be properly completed (malabsorption).

For celiac and other gluten‐related diseases, the only treatment method is a gluten‐free diet. The exact amount of gluten that individuals can tolerate can vary. However, even today, gluten cannot be completely eliminated in products labeled as “gluten‐free.” For this reason, it is very important to determine the gluten limits in gluten‐free products so that they do not cause harmful effects on celiac patients (Walter et al., 2014). The safe limit that will not cause possible discomfort is a daily gluten intake of less than 10 mg (Catassi et al., 2007). The Turkish Food Codex Communiqué on meals suitable for people with gluten intolerance (Communiqué No: 2012/4) states that 20 ppm of gluten is the maximum amount that can be present in gluten‐free meals (Eksin et al., 2015). The only reason the applied treatment fails is that gluten is not completely removed from the diet. Failure to fully comply with this treatment causes an increase in the morbidity and mortality rates (Biagi et al., 2012; Niewinski, 2008). Patients who have not been diagnosed, or who do not comply with a gluten‐free diet are prone to many complications (osteoporosis, infertility, autoimmune diseases, etc.). The rise in cancer mortality among celiac disease patients and their family is proof to the condition's clinical importance (Brousse & Meijer, 2005). To guarantee that celiac patients’ diets are safe, it is crucial to assess the purity of gluten‐free items. Verification of specificity, reproducibility, and reliability are necessary for a viable analytical approach to measure the gluten levels in foods (Scherf & Poms, 2016). For this purpose, certain analytical methods for detecting gluten were developed and are documented in the literature. Enzyme‐linked immunosorbent assays (ELISA) based on R5 and G12 antibodies are advised under the present regulations (Alvarez et al., 2013). ELISAs are widely used because of their sensitivity and specificity and their suitability for continuous analysis, as well as the absence of any other method that can be considered as an independent reference for gluten analysis. The development of novel immunosensors using techniques like aptamers, microarrays, and multi‐analyte profiling can offer an alternative to ELISA and several other analytical techniques (such as proteomics, mass spectrometry, and genomics). Some criteria in analytical methodologies are sought for the accurate measurement of gluten levels in foods. These criteria must have a high enough sensitivity, specificity, repeatability, robustness, and ability to be verified by experiments in multiple independent laboratories (Slot et al., 2015).

The sensitivity limit that must be met in the currently used analytical methods in gluten‐free product analysis should be 20 mg gluten/kg as reported by the Codex. Many of the ELISAs used are frequently preferred due to their specific structure, high sensitivity, and allowing routine analysis (Amigo & Popping, 2012). ELISAs continue to have several limitations, however, including influences from food matrix and processing history and unpredictability of the findings in current ELISA test kits for diverse assays, despite the introduction of novel specific antibodies for use in gluten analysis and several enhanced extraction procedures. An ideal combination of extraction operation and testing equipment should consider the challenges and factors specific to highly heat‐treated and hydrolyzed gluten. Polymerase Chain Reaction (PCR)‐based tests have also been known to have these complications (Gomaa & Boye, 2015; Sandberg et al., 2003). DNA‐based approaches can potentially provide better findings than ELISAs in terms of sensitivity and specificity, however they are not suited for gluten detection in severely processed or hydrolyzed materials with significant DNA degradation (Scharf et al., 2013). The most promising methods for accurately quantifying residues of gluten to assure the safety of gluten‐free diets for CD patients are liquid chromatography–mass spectrometry (LC–MS)/mass spectrometry (MS) separation methods because of their high selectivity, sensitivity, adaptability, and extensive application for heated and hydrolyzed gluten (Fiedler et al., 2014; Haraszi et al., 2011). However, due to the high cost of the equipment and the need for specialists, these methods are not commonly used in conventional analysis, but are instead used in specialized laboratories and research institutions. New methods aim to improve gluten analysis with simple, rapid, and in situ detection procedures that provide lower cross‐reactivity as well as better sensitivity with related species (Scherf & Poms, 2016). Electrochemical techniques are inexpensive, portable, easy to operate, and fast; therefore, they are often preferred over the other analytical techniques and have been shown to be useful tools for food detection (Amrutha et al., 2019; Edwin et al., 2021; Eksin et al., 2015; Manjunatha et al., 2009; Prinith et al., 2019; Raril et al., 2018).

In order to accurately and selectively detect gluten, Erdem et al. developed an electrochemical disposable pencil graphite electrode (PGE) in conjunction with the differential pulse voltammetry (DPV) technique. The linear concentration range for gluten was determined as between 20 and 100 µg/mL under ideal experimental circumstances, and the detection limit was 7.11 µg/mL (Eksin et al., 2015). Recently, a promising method for finding gluten in flours was reported, combining surface‐modified nanoparticles with electrochemical detection. So, utilizing the molecularly imprinted polymers (MIP) method, which enables the construction of a complex polymeric cavity with functional monomers using a template, a gluten‐sensitive receptor was developed. In this study, magnetic MIPs were employed to allow an external magnetic field to analytically detect an electrochemical signal. A detection limit of 8.5 mg/kg was determined after testing several wheat samples for gluten‐magnetic molecularly imprinted polymers (MMIP) (Limthin et al., 2019).

Electrodes play a very important role in electroanalytical techniques as they provide the interface between the analyte and the electrochemical cell. Modified electrodes have been applied in various fields such as energy production and analytical chemistry. Modified electrodes have been particularly successful in the electrochemical determination of trace levels of amino acids, peptides, proteins, alcohols, and sugars, but have also been used for the study of inorganic ions. Electrodes modified with nanomaterials have significantly increased the sensitivity of electroanalytical techniques because nanomaterials support the electrocatalysis process and increase the surface area of the electrode (Cox et al., 1996).

In this study, MoS_2_ nanoparticles, a promising two‐dimensional material with a wide range of potential applications due to its superior electrochemical, mechanical, and optical characteristics (Pietrzyk et al., 2018; Tian et al., 2014; Zhou et al., 2016), were synthesized using chemical synthesis. Detailed characterization (x‐ray diffraction [XRD], scanning electron microscopy [SEM], and x‐ray photoelectron spectroscopy [XPS]) was performed and the developed modified MoS_2_ nanoflowers/GCE electrode was subjected to the gluten test with electrochemical measurements and DPV (Tigari et al., 2020). Finally, MoS_2_ nanoflowers/GCE was successfully applied to a commercial sample and method validation was achieved with the ELISA method.

## 2 MATERIALS AND METHODS

### 2.1 Reagents and instrumentation

Sigma‐Aldrich supplied the high‐purity sodium molybdate dihydrate (Na_2_MoO_4_·2H_2_O, ≥99 %) as a source for molybdenum and the l‐cysteine (C_3_H_7_NO_2_S, 97 %) as a source for sulfur, which were both used in the preparation of the MoS_2_ nanoparticles. Additionally, gluten, ethanol, and deionized water, among other things, were purchased from Sigma‐Aldrich (St. Louis, MO, USA). All reagents were used without further purification.

Gluten ELISA kits were also acquired from Sigma‐Aldrich and the assay was carried out according to the manufacturer guidelines.

The crystal structure was observed with XRD (PANalytical, the Netherlands). The morphology of MoS_2_ was observed with a scanning electron microscopy with energy dispersive spectroscopy (SEM‐EDS, ZEISS GeminiSEM) and XPS (SPECS). Electrochemical measurements were made using a BioLogic SP‐50 potentiostat (BioLogic Company France).

### 2.2 Synthesis of MoS_2_ nanoparticles and electrochemical measurements

MoS_2_ nanoflowers were synthesized with a one‐step hydrothermal method (Liang et al., 2018). Briefly, 50 mg of Na_2_MoO_4_·2H_2_O and 100 mg of l‐cysteine were added to 25 mL of distilled water and stirred for 30 min at room temperature to form a homogenous suspension. A stainless‐steel autoclave coated with Teflon was then used to heat the suspension for 24 h at 220°C. The black material was then separated using centrifugation, washed with plenty of water and ethanol, and allowed to dry at 70°C overnight.

To form a catalyst ink, 5 mg of catalyst was dispersed in 1 mL of water–ethanol solution (volume ratio of 1:1) with 20 µL of Nafion solution (5 wt%) to form a homogeneous ink by sonication for 5 min. Before each experiment, the GCE was refined with slurries of smaller alumina sizes (1, 0.3, and 0.05 µm) followed by sonication for 5 min in purified water. The obtained modified MoS_2_ nanoflowers/GCE was tested with the DPV method by applying 20 mV pulse width conditions with 0 to 1.2 V scanning range and 50 mV/s scan rate for gluten detection by electrochemical analysis. For DPV measurements, a glassy carbon electrode with a diameter of 2 mm as a working electrode and a Pt wire and an Ag | AgCl electrode was used as reference electrode in 3 M KCl. In this study, determination of gluten was tested in vinegar and method validation was carried out with the ELISA method.

## 3 RESULTS AND DISCUSSION

### 3.1 Characterization

**FIGURE 1:**
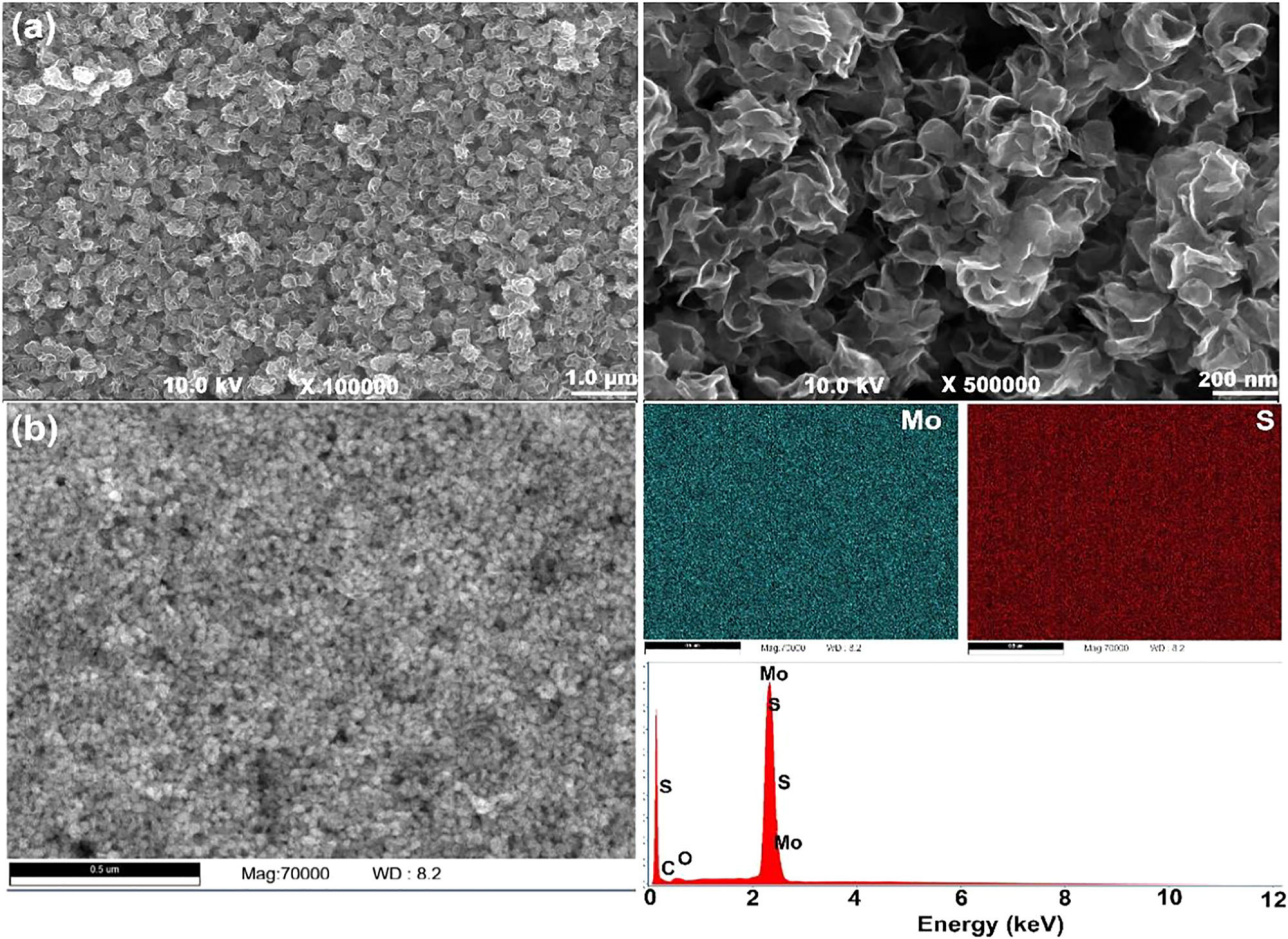
(a) Scanning electron microscopy (SEM) micrograph of MoS_2_ nanoflowers with low (left) and high (right) magnifications and (b) SEM‐elemental mapping of Mo and S elements and energy dispersive spectroscopy spectrum of MoS_2_ nanoflowers.

The structure and morphology of MoS_2_ nanoflowers were investigated using SEM and the micrographs of the sample are shown in Figure 1a. The samples have mean diameters of 230 nm and the high magnification SEM image showed that the nanoflowers comprise thin nanosheets with a mean diameter of 18 nm. In addition, SEM‐elemental mapping of nanoflowers showed a homogenous distribution of Mo and S elements and also recorded the same elements as the energy dispersive spectroscopy (EDS) spectrum of the sample (Figure 1b) indicating the successful synthesis of MoS_2_ nanoflowers.

**FIGURE 2:**
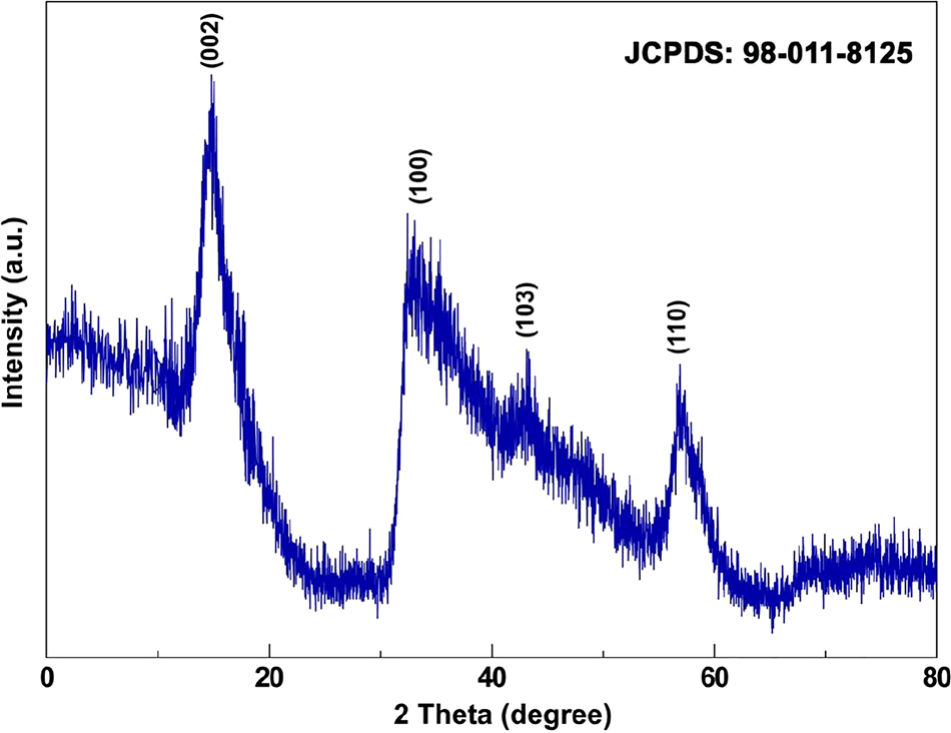
X‐ray diffraction pattern of MoS_2_ nanoflowers.

XRD was used to determine the crystallinity and crystal structure of MoS_2_ nanoflowers. The XRD pattern of the MoS_2_ nanoflowers (Figure 2) shows the main diffraction peaks recorded at 14.3°, 33.6°, 43.1°, and 58.6 °, which can be indexed as (002), (100), (103), and (110), respectively, which matched well with the hexagonal phase of MoS_2_ (JCPDS: 98‐011‐8125) (Liu et al., 2019).

**FIGURE 3:**
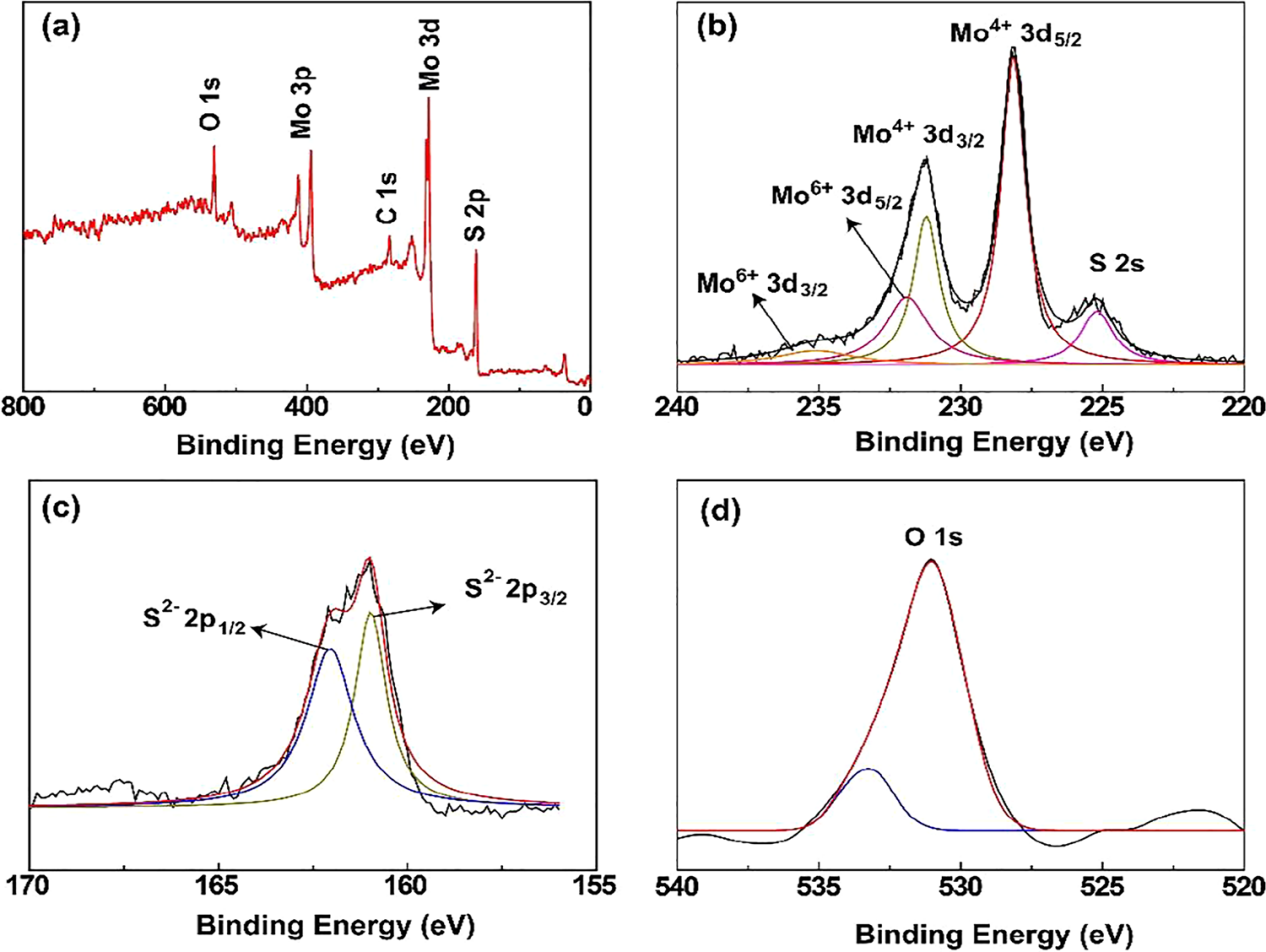
(a) X‐ray photoelectron spectroscopy (XPS) survey scan of MoS_2_ nanoflowers and XPS core‐level spectra of MoS_2_ nanoflowers for (b) Mo 3d, (c) S 2p, and (d) O 1s.

The chemical composition and oxidation states of the elements in MoS_2_ nanoflowers were determined by XPS. As displayed in Figure 3a, in the survey scan of MoS_2_ nanoflowers, the appearance of Mo and S clearly confirmed the presence of Mo and S. The appearance of weak C 1s can be attributed to the adsorption of carbonaceous contaminants during the XPS operation (Pu et al., 2015). In the core‐level spectra of Mo 3d (Figure 3b), the presence of Mo 3d doublet at about 228.1 eV (3d 5/2) and 231.2 eV (3d 3/2) indicate the typical 4+ oxidation state of Mo (Chang et al., 2013). In addition, the two different peaks of Mo^6+^ (231.9 eV for 3d 5/2 and 235.1 eV for 3d 3/2) were also observed, implying slight oxidation of MoS_2_ (Hai et al., 2016). In the core‐level spectra of S 2p (Figure 3c), distinct peaks were observed at 161.5 eV (S 2p 3/2) and 162.8 eV (S 2p 1/2), respectively, which supported S^2−^ binding energies in 2H‐MoS_2_ (Oakes et al., 2016). Moreover, the core level of O 1s spectra (Figure 3d) can be deconvoluted into two peaks at 531.7 and 532.9 eV, attributed to O^2−^ species which are most probably due to the absorption of substances containing oxygen, such as water (Huang et al., 2020).

### 3.2 Electrochemical characterization of gluten

**FIGURE 4:**
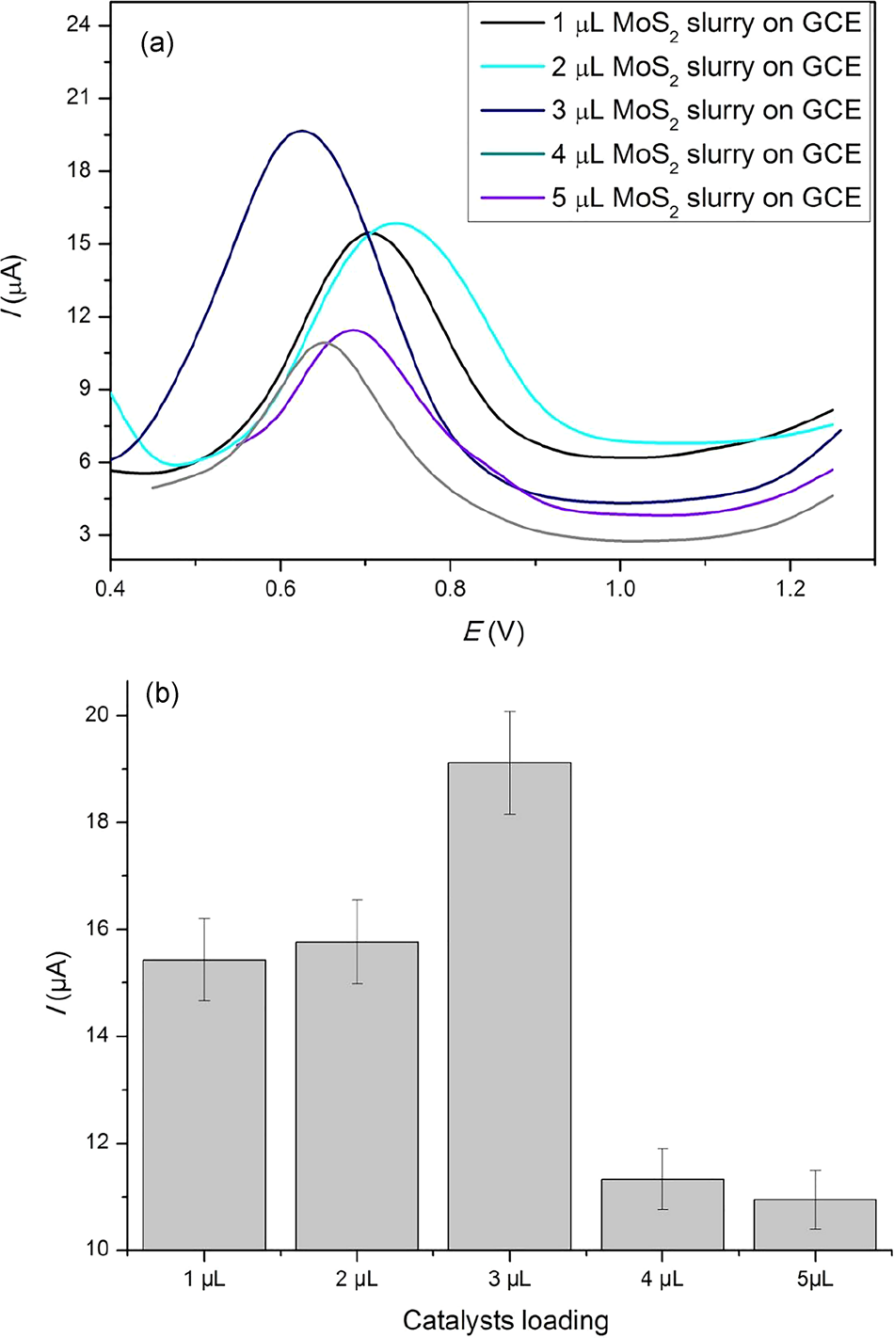
(a) Differential pulse voltammetry of the MoS_2_ loading amount on the GCE surface. (b) Column graph of the catalyst loading amount (reaction conditions; pulse width: 20 mV, scan rate: 50 mV/s, 20 ppm gluten, 5 mg MoS_2_ in 0.1 M PBS, pH 4.5 at room temperature).

The thickness of a flat catalyst layer on a solid substrate determines its diffusion state (Du et al., 2017). Therefore, to investigate the impact of diffusion limitation in catalysts, electrodes must be produced with various catalyst layer thicknesses. The MoS_2_ catalyst slurry was obtained from 5 mg MoS_2_ and 1 mL (Nafion + ethanol). The effect of catalyst loading on the electrode (MoS_2_ nanoflowers/GCE) surface in the presence of 20 µg/mL (ppm) gluten dissolved in dimethyl sulfoxide (DMSO) in the medium was transferred to 1, 2, 3, 4, and 5 µL and examined in 0.1 M phosphate buffered saline (PBS) buffer (pH 4.5). The gluten detection performance of the catalyst loading was studied using the DPV method at 20 mV pulse width and 50 mV/s scan rate (Figure 4a) and the results are plotted on a bar graph (Figure 4b). Instead of cyclic voltammetry, DPV was used, as this method offers better sensitivity and signal‐to‐noise ratio (Salman et al., 2020). At 1–2 µL thickness, the gluten current is slightly increased (from 15 to 16 µA). With a thickness of 3 µL, the detection current increased to 19 µA. In addition, the above selectivity was reduced to 10 µA in the case of a thicker catalyst layer (4–5 µL).

The scan rate controls how fast the applied potential is scanned, and faster scan rates lead to a reduction in the size of the diffusion layer, resulting in higher currents being observed.

In our study, the active surface area of the electrode produced using the cyclic voltammetry (CV approach was determined. For electrochemically reversible electron transfer processes involving freely diffusing redox species, the Randles–Sevcik equation (Equation 1) describes how the peak current ip (A) increases linearly with the square root of the scan rate *υ* (V/s).

For this purpose, the electrochemical active surface area (A) in a 5 mM [Fe(CN)_6_]^3−/4−^ solution containing 0.1 M KCl was measured by CV tests at different scanning speeds.

**FIGURE 5:**
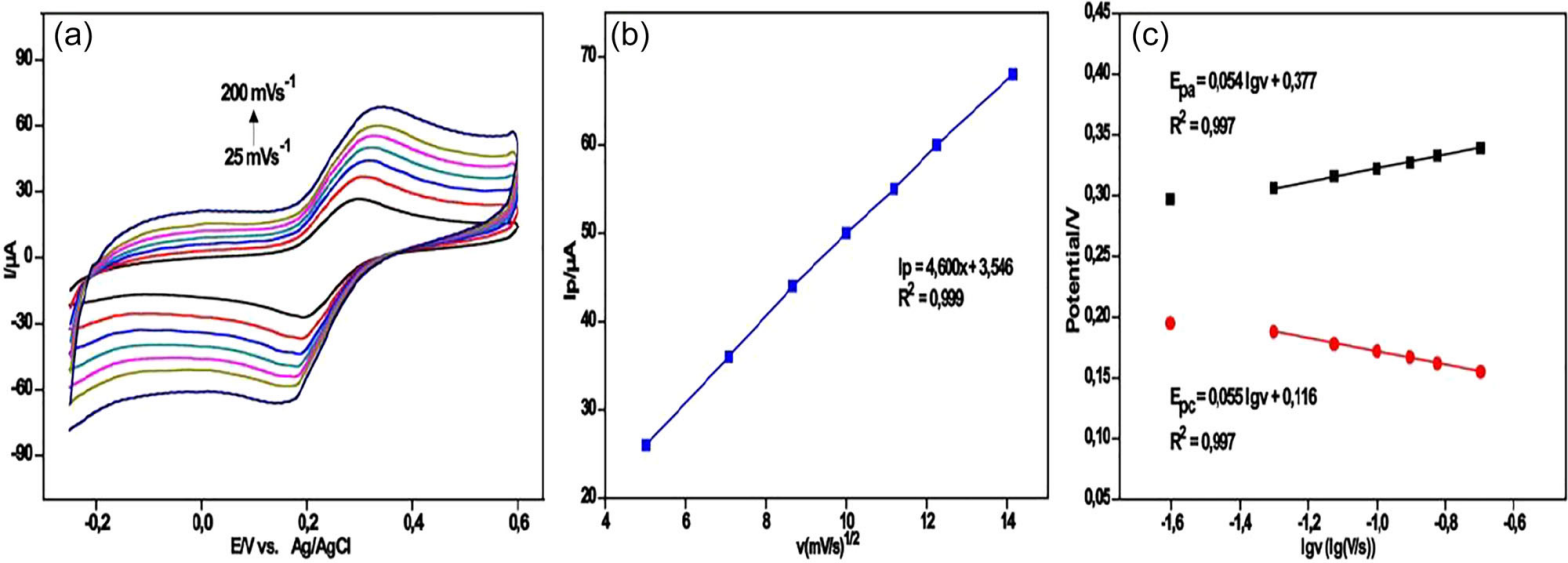
(a) CV of MoS_2_/GCE in 5 mM [Fe(CN)_6_]^3−/4−^ containing 0.1 M KCl at different scan rates. (b)The linear relationship between the positive and negative peak current and the square root of the scanning rate. (c) The relationship between the positive and negative potential of the peak and the logarithm of the scanning rate.

As shown in Figure 5a, both anode and cathode peak currents increase with the increase of scanning speed, indicating that the electrode reaction of the process is diffusion controlled. In a study examining the methane selectivity with cobalt‐based monolithic catalysts in the literature, monolithic catalysts with layers greater than 50 µm were reported to exhibit a significantly reduced methane selectivity and were observed to suffer from diffusion limitations (Guettel et al., 2008).

(1) $$\mathrm{ip}=\left(2.69\times 10^{5}\right)n^{3/2}D^{1/2}A\cdot^{1/2}c$$

where “*c*” is the concentration of K_3_[Fe(CN)_6_] (*c* = 5.0 mM) and “*A*“ represents the electrochemically active substance area, “*n*” is the number of electrons, “*ʋ*” is the scan rate, and “*D*” is the diffusion coefficient (6.7 × 10^6^ cm^2^/s^1^). In Figure 5, the electrochemical active surface area of MoS_2_ nanoflowers/GCE is 0.082 cm^2^ (0.053 cm^2^), which is almost twice that of the bare electrode. It is responsible for the enhanced detection sensitivity.

**FIGURE 6:**
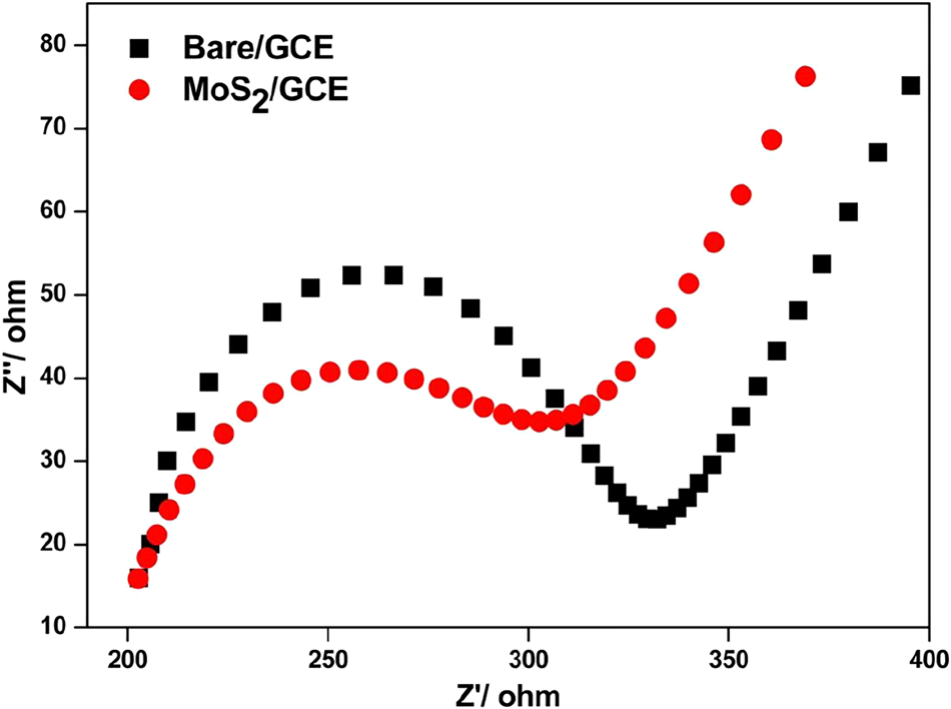
Electrochemical impedance spectroscopy spectra recorded on bare GCE and MoS_2_/GCE in 5.0 mM [Fe (CN)_6_]^3−/4−^ solution. Scan rate: 50 mV/s.

Electron transfer between the electrolyte and electrode surfaces was elucidated by the electrochemical impedance spectroscopy (EIS) method. A typical EIS spectrum contains a higher frequency semicircle and a lower frequency linear region. The semicircular part corresponds to the electron transfer control process in the spectrum, and the linear part corresponds to the diffusion control process. The diameter of the semicircle is equivalent to the electron transfer resistance value (R_ct_) of the electrode surface, and generally Rct is smaller and the interfacial electron transfer rate is higher for the smaller semicircle diameter. The EIS results for the bare electrode and MoS_2_/GCE are shown in Figure 6. MoS_2_/GCE showed the smallest Rct, and this finding indicates that the electrode fabricated with MoS_2_ has a strong electron conduction path between the electrode and the electrolyte.

With the purpose of further understanding the electrochemical reaction kinetics of the sensor, the charge transfer coefficient (*α*) was calculated using Laviron's formula (Equation 2):

(2) $$lg\;\frac{Ka}{Kc}=\;lg\frac{\alpha }{1-\alpha }$$

In the above equations, *Kα* and *Kc* are the slopes of the linear relationship between the peak potential and the logarithm of the scan rate in Figure 5c. According to theoretical calculation, *α* is 0.99. The higher electron mobility proves the outstanding electrical conductivity and excellent sensing potential of MoS_2_/GCE.

In electroanalysis, electrode–solution coupling is of vital importance because the maintenance of a diffusion curve causes analytes to migrate from the entire solution approach to the electrode surface in the absence of applied voltage, and the concentration of the analyte across the surface of the electrode can influence a number of electrochemical variables (Kazici et al., 2019).

**FIGURE 7:**
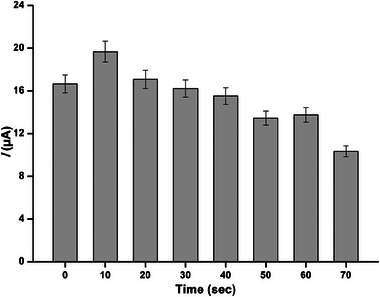
Amount of accumulating time's effect on the 20 ppm Glu electrochemical study at a scan rate of 50 mV/s at various time intervals between 0 and 70 s.

In the current study, while examining the effect of accumulation times between 0 and 70 s at various time points to investigate the effect of concentration in the region, it was concluded that analyte saturation was reached at this time, as 10 s provided the highest peak current. This optimized time tactic was applied in subsequent trials (Figure 7).

**FIGURE 8:**
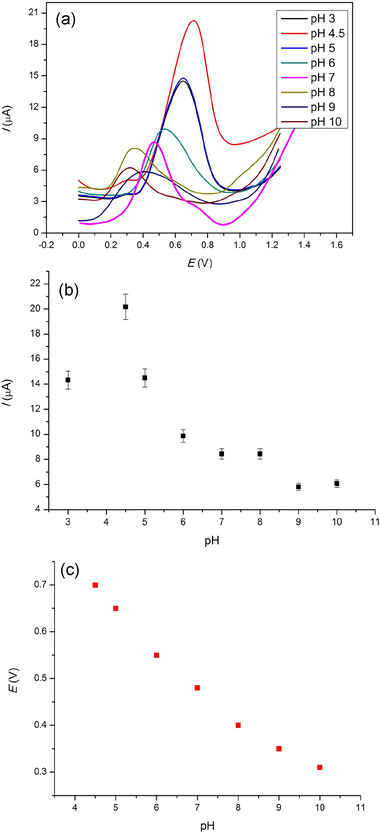
(a) Differential pulse voltammetry gluten detection on the MoS_2_ nanoflowers/GCE surface in different pH solutions. (b) Column graph of pH. (c) The peak potential versus pH value (reaction conditions; pulse width: 20 mV, scan rate: 50 mV/s, 20 ppm, 5 mg MoS_2_, 3 µL MoS_2_ slurry on GCE in 0.1 M PBS, pH 3–10 at room temperature).

The current team kept working on improving the pH of the supporting electrolyte and developing an analytical method for gluten detection. The effect of pH values of working solutions was investigated in the range from 3 to 10.0 using 0.1 M PBS solution. Experimental results are given by the DPV method (Figure 8a) and bar graph (Figure 8b). In the present study, a significant decrease in gluten detection current was recorded after pH 4.5. Gluten is a protein family found in wheat, rye, oats, and barley, which can cause health problems in people who are sensitive to it (Scherf & Poms, 2016). While mild pH (4.5) treatment provides high detection current by causing higher protein solubility, higher pH (10) may have reduced the detection current by causing damage to the structure and denaturation of proteins (Shan et al., 2012). In addition, the pH value was examined in the range of 3–10, as seen in Figure 8a,b, but the range of 4.5–10 was examined to examine the linear change of its change with potential. It is obvious that the current decreases at lower pH values. As pH increases, the peak potential (E_pa_) decreases proportionally and becomes linear as shown in Figure 8c. This is attributed to protons participating in the electrochemical reaction process.

The linear regression equation between Epa and pH is Epa (V) = 1.0006 – 0.072 pH (*R* ^2^ = 0.98); from here the absolute value of the slope (0.072 V/pH) is close to the theoretical Nernstian value: 0.059 V/pH means a fairly equal number of electrons and protons involved in the redox reaction process.

In electroanalysis, electrode–solution coupling is of vital importance because the maintenance of a diffusion curve causes analytes to migrate from the entire solution approach to the electrode surface in the absence of applied voltage, and the concentration of the analyte across the surface of the electrode can influence a number of electrochemical variables (Shufang et al., 2022).

### 3.3 DPV‐based non‐enzymatic gluten detection by using the MoS_2_ nanoflowers/GCE electrode

To evaluate the electroanalytical efficacy of the MoS_2_ nanoflowers/GCE, gluten detection was carried out under the optimized conditions mentioned above. Again, the DPV method was used, as it is a useful tool for the swift and precise detection of analytes in electroanalytical chemistry due to structural high current sensitivity and low charge contribution to background current (Li et al., 2019).

**FIGURE 9:**
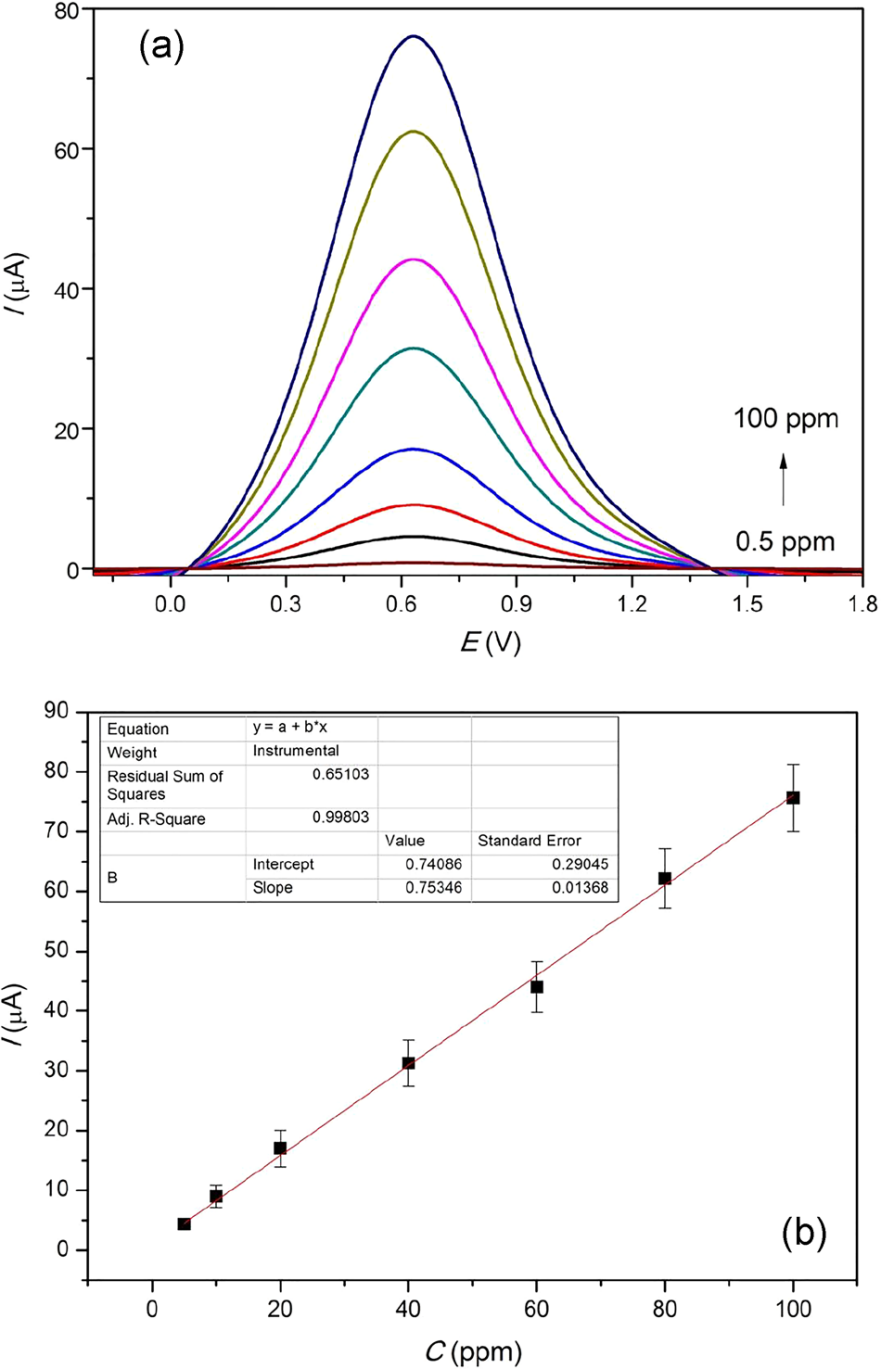
(a) Differential pulse voltammetry curves of the MoS_2_ nanoflowers/GCE electrodes fabricated with different gluten concentrations (from 0.5 to 100 ppm) in pH 4.5 PBS. (b) Relationship between the peak currents and concentrations.

Figure 9a depicts the DPV curves of MoS_2_ nanoflowers/GCE for determining different concentrations of gluten in PBS solution (0.1 M, pH = 4.5). Gluten concentrations were 0.5, 1, 5, 10, 20, 40, 60, 80, and 100 ppm, respectively. The scan range was between 0 and 1.2 V, amplitude 50 mV, pulse duration 0.5 s, and potential increase 4 mV. As shown in the figure, when the analyte concentration increases, the peak value of the response currents increases, indicating that an efficient electron transfer takes place between the gluten and modified electrode (MoS_2_ nanoflowers/GCE). The oxidation peak potentials of gluten on these MoS_2_ nanoflowers/GCE sensor fabricated at various gluten concentrations were stable at around 0.6 V. The linear relationship between gluten (*C*) concentration and response currents (*I*) is displayed in Figure 7b. $I\;\left(\mu A\right)=\;0.75346C_{\mathrm{gluten}}\left(\mathrm{ppm}\right)+0.74086,\;\;R^{2}=\;0.99$ was the linear equation obtained for 0.5–100 ppm. The quantity limit (LOQ) and the detection limit (LOD) of the current assay were computed as follows: detection limit (LOD) = 3*σ*/*S* and quantity limit (LOQ) = 10*σ*/*S*; where *σ* is the standard deviation and *S* is the slope of the calibration curve. LOD and LOQ values were found as 0.16 and 0.57 ppm, respectively. According to the Turkish Food Codex regulation, the gluten LOD is substantially lower (less than 20 ppm) than the permissible amount for gluten‐free branded items (Eksin et al., 2015). In terms of linear range and LOD, the suggested sensor's responses were also contrasted with those of the previous gluten sensors. The findings are given in Table 1. The sensor in this study has the lowest detection limit among the other electrochemical gluten sensors, a satisfactory linear range among highly limited electrochemical gluten sensors in the literature, as well as superior properties in comparison to the other expensive methods in terms of cheapness and ease of use.

**TABLE 1:** Overview of gluten analysis methods designed to detect gluten values of 20 mg/kg or less, sorted first by method type and then by linear volume range and sensitivity and reference number.

Method	Linear range	Sensitivity	Ref.
ELISA	–	Antibodies; G12 (LOQ: 2.9 ± 0.3 µg/mL), 2D4 (LOQ: 2.8 ± 0.2 µg/mL), Skerritt (LOQ: 0.85 ± 0.04 µg/mL)	Panda et al. (2017)
ELISA	–	LOQ: 5 mg/kg of gluten	Lacorn et al. (2019)
Genomics, PCR	–	LOD: 0.9 mg/kg of gluten	Ahmed and Meng (2019)
Genomics, PCR	–	LOD: 1–5 mg/kg of gluten	García‐García et al. (2019)
Sensor, aptamers (GE‐Cys‐PAMAM‐Gli1)	–	LOD: 5 mg/kg of gluten	Malvano et al. (2017)
Sensor, aptamers, and G12 and barley antibodies	–	LOD: 0.1–1.0 µg/mL of gluten	White et al. (2018)
DNA aptamers	0.075–100 µM	LOD: 0.5 µg/mL	Amaya‐Gonzalez et al. (2014)
Iridium complex Phosphorescent Probe	5–200 µg/mL	LOD: 2.6 µg/mL	Huang et al. (2018)
Gold nanoparticles‐light Scattering	1.5–15 µg/mL	LOD: 0.8 µg/mL	Molina‐Delgado et al. (2011)
Sensor, MMIP	20–1000 µg/mL	LOD: 8.5 mg/kg of gluten‐ MMIP	Limthin et al. (2019)
Sensor, PGE	20–100 µg/mL	LOD: 7.11 µg/mL of gluten	Eksin et al. (2015)
Sensor, MoS_2_ nanoflowers/GCE	0.5–100 µg/mL	LOD:0.16 and LOQ:0.57 µg/mL of gluten	This study

Abbreviations: GE, gold electrode; MMIP, magnetic molecularly imprinted polymers; PGE, pencil graphite electrode.

The sensitivity of the sensor was evaluated as 17.92 µA mL/µg cm^2^ (slope divided by the electrode surface area).

Recently, different ELISA and enzyme‐linked oligonucleotide assay (ELONA) formats that may identify gluten proteins have been developed to test the gluten content of foods (Arendt et al., 2008). All sensors have molecules (such as antibodies–aptamers) that act as receptors fixed on the surface of the substrate. Following antigens binding, an enzymatic process occurs that produces fluorescent or electrochemical signals. For the amperometric sensor, a gold‐layered working electrode, Ag/AgCl‐layered reference electrode, and counter electrode were attached to a chip surface. With this study, we can report that the functionality of these chip systems is promising.

### 3.4 Specificity study

**FIGURE 10:**
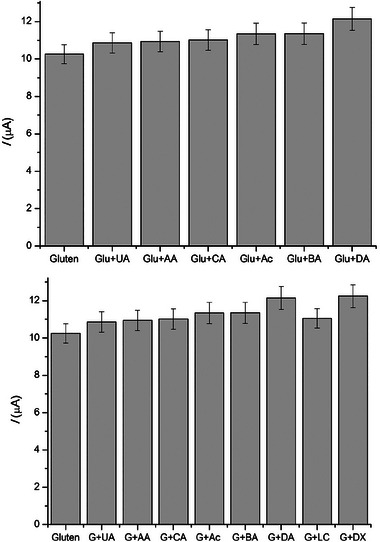
Interfering effect of 10 ppm (uric acid, ascorbic acid, citric acid, acetic acid, benzoic acid, and dopamine) on the bar graph of differential pulse voltammetry response of MoS_2_ nanoflowers/GCE upon the successive addition of 10 ppm of gluten in 0.1 mM PBS, pH 4.5.

In addition, interference experiments were also conducted to determine the effect of impurities such as organic acids on the current density of gluten (Figure 10). According to the literature, gluten can be oxidized simultaneously with some substances (such acetic acid [Ac], uric acid [UA], ascorbic acid [AA], dopamine [DA], citric acid [CA], benzoic acid [BA], dextrose [DX], and lactose [LC]) and contribute to the interference signals (Su et al., 2011). Figure 10 presents the selectivity test results for 10 ppm of gluten with 10 ppm UA, AA, CA, Ac, BA, DA, DX, and LC, respectively. It was found that all these elements produced insignificant current responses and therefore, the MoS_2_ nanoflowers/GCE had excellent specificity and anti‐interference ability for some interfering species.

### 3.5 Reproducibility, stability, repeatability, and durability assay

**FIGURE 11:**
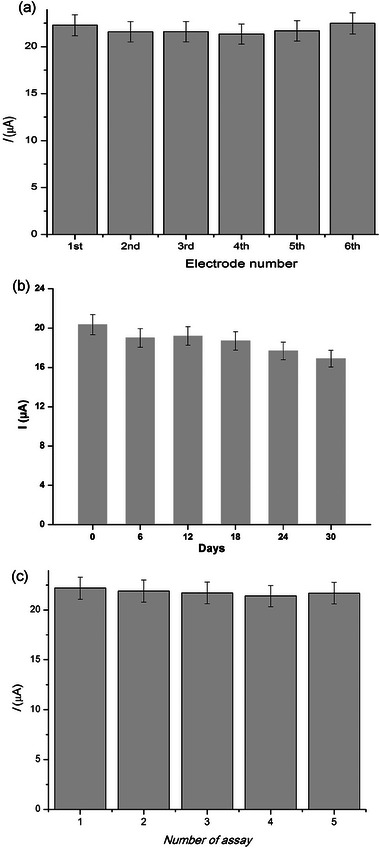
(a) The reproducibility of MoS_2_ nanoflowers/GCE electrode for gluten detection. (b) Peak current of CV versus storing time for MoS_2_ nanoflowers/GCE in 20 ppm of gluten in 0.1 mM PBS, pH 4.5 at 50 mV/s. (c) Differential pulse voltammetry peak current versus number of assay in five 10 ppm of gluten in 0.1 mM PBS, pH 4.5 at 50 mV/s using one MoS_2_ nanoflowers/GCE electrode.

Reproducibility was also investigated as shown in Figure 11a. Six sensors prepared in the same batch were examined in pH 4.5 PBS individually. The relative standard deviation (RSD) of the response currents was 2.03%, demonstrating that the improved sensor showed excellent performance in terms of **reproducibility**.

In addition, stability of the MoS_2_ nanoflowers/GCE sensor was investigated to determine the activity of the sensor in practical use (Figure 11b). The fabricated electrode was stored in an airtight container for 30 days to ensure optimum reliability and stability over time at 4°C for, and as a result of the measurement at the end of the 12th day, 94% remained and at the end of the 30th day, 83.8% remained. This indicates that the prepared MoS_2_ nanoflowers/GCE sensor has good **stability** (Figure 11b).

A daily evaluation was performed to verify that measurements were repeatable and prepared MoS_2_ nanoflowers/GCE electrode was used for four parallel detections, and the relative standard deviation RSD was 1.54%, indicating that the **repeatability** of the MoS_2_ nanoflowers / GCE sensor was very good (Figure 11c)

**FIGURE 12:**
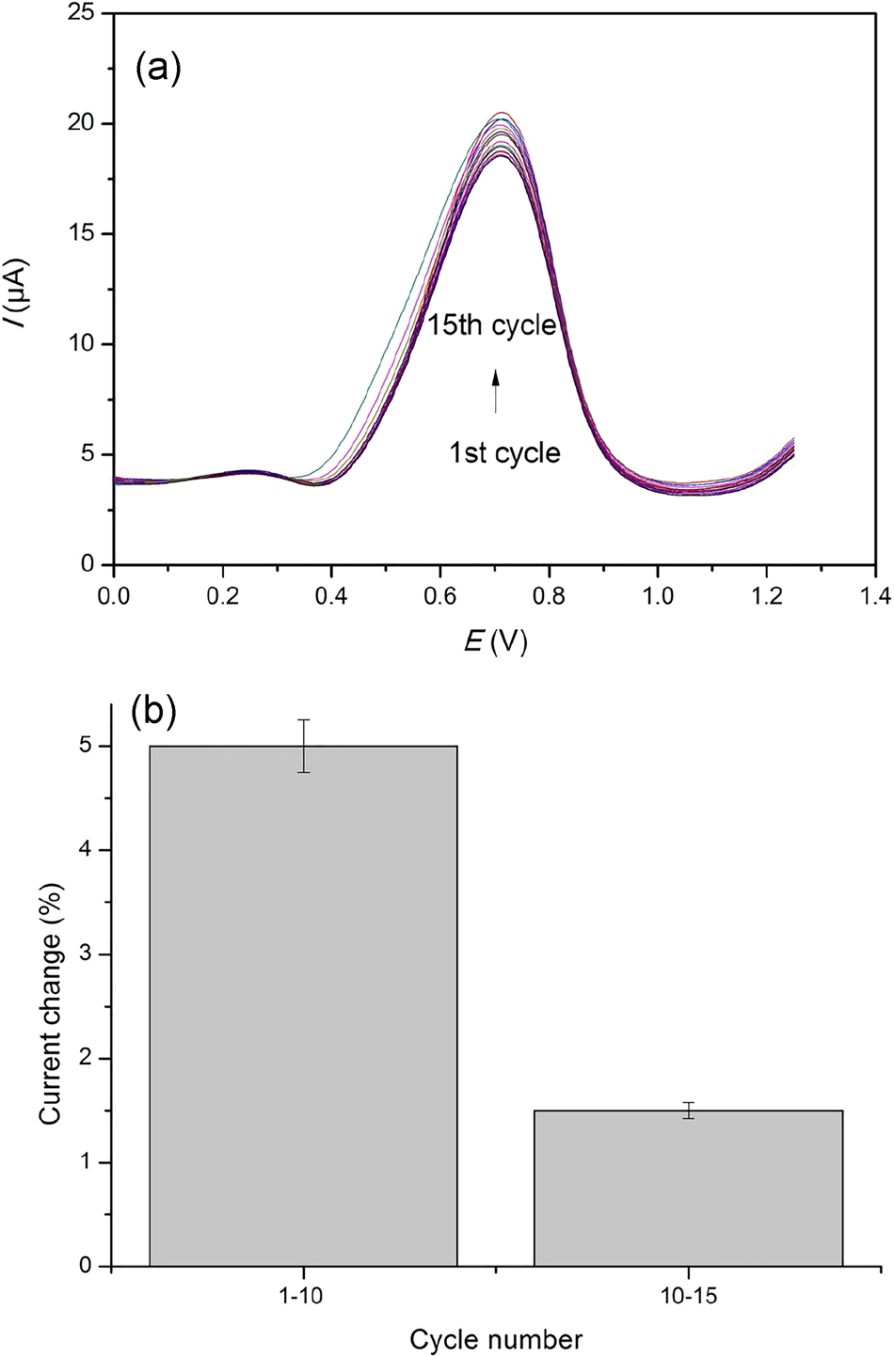
(a) Effect of the number of cycles on the differential pulse voltammetry gluten detection on the MoS_2_ nanoflowers/GCE surface. (b) Column graph of cycle number against the percentage of current change (reaction conditions; pulse width: 20 mV, scan rate: 50 mV/s, 20 ppm gluten, 5 mg MoS_2_, 3 µL MoS_2_ slurry on GCE in 0.1 M PBS, pH 4.5 at room temperature).

In addition, the durability of the sensing system was examined by measuring the electrochemical response of gluten at the modified electrode in the presence of 20 ppm gluten for 15 cycles (Figure 12a). From these consecutive cyclic runs, the % RSD of peak current was calculated as 4.32%. Up to the first 10 cycles, the current change was 5%. The current change percentage of the developed electrode decreased to 1.5% after 10 cycles and stabilized (Figure 12b).

### 3.6 Determination of gluten in commercial sample

To further investigate the practical application of the developed gluten sensor, different gluten concentrations were detected in vinegar samples. The reliability of the produced electrode was examined by a recovery test and for this purpose, the resulting vinegar samples had gluten added for detection in 0.01 M PBS (pH = 4.5) medium. Each sample was tested in triplicate and the average recoveries are given in Table 2. The results demonstrate that the gluten concentration is very close to the concentration of the gluten standard solution in the real sample and the recovery range and RSD values are 98%–99.7% and 4.13%–5.03%, respectively, with the proposed method. To confirm the amount of gluten with the proposed test, the gluten concentration in the vinegar sample described above was obtained using ELISA analysis (Panda et al., 2017). The gluten concentration obtained in the real sample showed good agreement between the recommended and ELISA methods, for both recovery (99.5%–100.5%) and standard error (2.10%–3.64%) values. Recovery rates and RSD values indicate that the proposed sensor (MoS_2_ nanoflowers/GCE) may be a promising candidate for quantitative detection of gluten.

**TABLE 2:** Comparison of the proposed method and enzyme‐containing immunosorbent assay method for recovery of gluten in spiked vinegar samples (*n *= 3) and dilution factor (1:10).

	Proposed method			ELISA method		
Added gluten (µg/mL)	Found (µg/mL)	Recovery^a^ (%)	RSD%	Found (µg/mL)	Recovery^a^ (%)	RSD%
0	–	–	–	–	–	–
2	1.96 ± 0.081	98.0	4.13	1.99 ± 0.055	99.5	2.76
4	3.94 ± 0.178	98.5	4.52	4.02 ± 0.121	100.5	2.76
6	5.94 ± 0.299	99.0	5.03	6.01 ± 0.211	100.2	3.51
8	7.97 ± 0.336	99.6	4.22	8.02 ± 0.292	100.3	3.64
10	9.97 ± 0.479	99.7	4.80	10.1 ± 0.212	100.1	2.10

^a^ Recovery (%) = [(Total)—(the measured concentration in real sample)] / spiked × 100.

## 4 CONCLUSIONS

Herein, we present a simple and precise enzyme‐free electrochemical gluten sensor based on the GCE electrode modified with MoS_2_ nanoflower particles. First, we performed the surface characterization of MoS_2_ nanoflower particles, and then we completed various optimization studies (such as the number of droplets on the surface and pH) for gluten detection using catalysts obtained by dropping these particles onto the GCE surface with an electrochemical technique. The application of gluten determination was tested with the DPV method, and the detection limit was obtained as 0.16 ppm which is in accordance with the official limits described in the Codex Standard. Also, this technique is highly selective with a wide concentration range of 0.5–100 ppm. In addition, the ability of this sensor to detect a mixture was studied, and to this end, different interventions, including dopamine, show that the sensor's ability to detect gluten in complex matrices is excellent. This electrochemical detection protocol provides fast, inexpensive, direct, sensitive, and selective determination of gluten without any external separation or using expensive biomolecules such as antibodies, DNA, or aptamers. This technique is a candidate to be a potential approach for other molecular detection methods.

## AUTHOR CONTRIBUTIONS

**Fırat Salman**: Formal analysis; data curation; validation. **Adem Zengin**: Conceptualization; writing—review and editing; investigation; supervision. **Hilal Çelik Kazici**: Conceptualization; investigation; funding acquisition; writing—original draft; methodology; writing—review and editing; supervision.

## CONFLICT OF INTEREST STATEMENT

The authors declare that they have no known competing financial interests or personal relationships that could have appeared to influence the work reported in this paper.
